# A steric gate prevents mutagenic dATP incorporation opposite 8‐oxo‐deoxyguanosine in mitochondrial DNA polymerases

**DOI:** 10.1111/febs.70064

**Published:** 2025-03-12

**Authors:** Noe Baruch‐Torres, Carlos H. Trasviña‐Arenas, Alexandru Ionut Gilea, Upeksha C. Dissanayake, Missael Molina‐Jiménez, Paola L. García‐Medel, Corina Díaz‐Quezada, Tiziana Lodi, G. Andrés Cisneros, Enrico Baruffini, Luis G. Brieba

**Affiliations:** ^1^ Unidad de Genómica Avanzada Centro de Investigación y de Estudios Avanzados del IPN (CINVESTAV‐IPN) Irapuato Mexico; ^2^ Department of Biochemistry and Molecular Biology University of Texas Medical Branch Galveston TX USA; ^3^ Sealy Center for Structural Biology and Molecular Biophysics Galveston TX USA; ^4^ Centro de Investigación sobre el Envejecimiento Centro de Investigación y de Estudios Avanzados (CINVESTAV‐IPN) Unidad Sede Sur Mexico City Mexico; ^5^ Department of Chemistry, Life Sciences and Environmental Sustainability University of Parma Italy; ^6^ Department of Chemistry and Biochemistry University of Texas at Dallas Richardson TX USA; ^7^ Department of Physics University of Texas at Dallas Richardson TX USA

**Keywords:** 8‐oxo‐deoxyguanosine, DNA polymerase, kinetic assay, mitochondrial DNA, ROS, translesion DNA synthesis

## Abstract

Reactive oxygen species (ROS) generate DNA lesions that alter genome integrity. Among those DNA lesions, 7,8‐dihydro‐8‐oxo‐2′‐deoxyguanosine (8‐oxodG) is particularly mutagenic. 8‐oxodG efficiently incorporates deoxycytidine monophosphate (dCMP) and deoxyadenosine monophosphate (dAMP) via base pairing mediated by its *anti* and *syn* conformations, respectively. In family‐A DNA polymerases (DNAPs), the amino acids responsible for modulating dCMP or dAMP incorporation across 8‐oxodG are located in a determined structural position. Those residues are a conserved tyrosine located at the N terminus of the α‐helix O and a nonconserved residue located six amino acids after this conserved tyrosine. In yeast mitochondrial DNAP (DNA‐directed DNA polymerase gamma MIP1 [Mip1]), those residues correspond to amino acids Y757 and F763. We hypothesized that the phenyl group of the F763 residue impinges on the *syn* conformation of 8‐oxodG, therefore reducing dAMP misincorporation. Here, we measured dCMP and dAMP incorporation across 8‐oxodG using wild‐type and F763 *Mip1* mutants. Our data suggest that both residue F763 and the universally conserved Y757 assemble a steric gate that obtrudes the 8‐oxodG(*syn*) conformation. As the human orthologue of Mip1, DNA polymerase gamma (HsPolγ) or DNAP γ, also harbors phenylalanine at the corresponding position to Mip1‐F763, the steric gate mechanism might similarly be responsible for controlling HsPolγ's fidelity when tolerating 8‐oxodG lesions.

## Graphical abstract

Reactive oxygen species induce mutagenic DNA lesions, such as 8‐oxo‐2′‐deoxyguanosine (8‐oxodG), which acts as a template for dCMP and dAMP incorporation through its anti and syn conformations. Specific residues must regulate the fidelity of templated 8‐oxodG. Our findings demonstrate that in yeast mitochondrial DNAP, residues F763 and Y757 form a steric gate that blocks the syn conformation of 8‐oxodG, ensuring high fidelity. A similar mechanism may be employed by the human orthologue, DNAP γ.

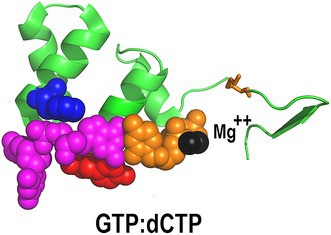

**Abbreviations**
8‐oxodG: 7,8‐dihydro‐8‐oxo‐2′‐deoxyguanosine
adPEO: Progressive External Ophthalmoplegia
BER: Base Excision Repair
BSA: bovine serum albumin
dAMP: deoxyadenosine monophosphate
dCMP: deoxycytidine monophosphate
DNAP: DNA polymerase
IMAC: immobilized metal affinity chromatography
ROS: reactive oxygen species
SASA: Solvent Accessible Surface Area
SC: synthetic complete
VMD: visual molecular dynamics

## Introduction

The mitochondrial theory of aging postulates that the accumulation of mitochondrial mutations leads to a gradual impairment in its function [1, 2, 3]. This theory implies that reactive oxidative species (ROS), which are a by‐product of respiration, are the main factors responsible for DNA damage. This generates a vicious cycle in which the damaged DNA alters oxidative phosphorylation proteins, thereby increasing the production of ROS One of the most common DNA lesions produced by ROS is the 7,8‐dihydro‐8‐oxo‐2′‐deoxyguanosine (8‐oxodG) as a cause of guanosine oxidation [4]. Guanosine oxidation at the C_8_ carbonyl group allows base pairing with dAMP via its *syn* conformation [5]. Thus, 8‐oxodG is a miscoding DNA lesion through its ability to efficiently template for dCTP and dATP [5]. Furthermore, the 3′‐5′ exonuclease proofreading domain of replicative DNA polymerases (DNAPs) is unable to detect an 8‐oxodG(*syn*):dAMP base pair as a mismatch [6, 7, 8]. DNAPs have different preferences for dATP incorporation when encountering an 8‐oxodG lesion [9, 10, 11, 12]. Thus, mutagenic incorporation occurs at the insertion step opposite 8‐oxodG.

Yeast and animal mitochondrial DNAPs are evolutionarilyrelated to DNAPs from T‐odd bacteriophages [13]. Residue K536 in bacteriophage T7 DNAP controls the ratio of mutagenic dAMP incorporation opposite 8‐oxodG [6, 14]. This residue avoids the formation of a Hoogsteen 8‐oxodG(*syn*):dATP base pair by means of unfavorable steric interactions and by promoting the formation of a Watson‐Crick 8‐oxodG(*anti*):dCTP base pair by establishing a hydrogen bond with the 8‐oxo group [6, 14]. In human (HsPolγ) and yeast (Mip1) mitochondrial DNAPs, the residues that correspond to T7 DNAP‐K536 are F961 and F763, respectively, suggesting that mitochondrial DNAPs favor dCMP incorporation opposite 8‐oxodG only by steric interactions that avoid the formation of an 8‐oxodG(*syn*):dATP base pair. Furthermore, HsPolγ and T7DNAP misincorporate dAMP opposite 8‐oxodG in approximately 10% and 33% of their replication events [6, 15]. The latter suggests that the bulky character of HsPolγ‐F961 is more efficient than the linear chain of four carbons and an epsilon amino group of lysine in T7DNAP‐K536 in obtruding the 8‐oxodG(*syn*) conformation. Interestingly, mutation of HsPolγ‐F961 (F961S) is observed in patients with autosomal dominant Progressive External Ophthalmoplegia (adPEO) [16].

In all family‐A DNA polymerases, a conserved tyrosine located in the α‐helix O of the finger subdomain assembles the DNAP active site upon binding the incoming nucleotide [17, 18, 19, 20, 21]. The identity of this universally conserved residue is Y757 and Y955 in Mip1 and HsPolγ, respectively. A Y955C mutation in HsPolγ decreases nucleotide incorporation fidelity and exhibits low catalytic efficiency [15, 22]. The HsPolγ‐Y955C mutation also increases dATP misincorporation across 8‐oxodG [15]. This mutation is also associated with adPEO, mitochondrial deletions, Parkinsonism, ovarian failure, and myopathy [15, 22, 23, 24, 25, 26, 27].

Yeast is a convenient system to study the pathogenic variants of HsPolγ [28, 29, 30]. Yeast mitochondrial DNAP, Mip1, is an intrinsically processive enzyme that does not need accessory proteins for full activity [31] and, like its human counterpart, uses RNAs transcribed by yeast mitochondrial RNA polymerase as primers [32, 33]. Graziewicz and coworkers proposed that residues Y955 and F961 of HsPolγ assemble a steric gate that impinges dATP incorporation opposite 8‐oxodG [15]. However, only the role of HsPolγ‐Y955 was experimentally demonstrated [15]. The corresponding residue to HsPolγ‐F961 in Mip1 is also phenylalanine (Mip1‐F763), suggesting that structure–function studies in Mip1 could be used to understand the mechanism that governs nucleotide incorporation across 8‐oxodG in mitochondrial DNAPs. Thus, here we use Mip1 to evaluate the role of residues Mip1‐F763 and Mip1‐Y757 on dNMP incorporation opposite an 8‐oxodG‐containing template.

## Results

### Residues HsPolγ‐F961 and Mip1‐F763 are structurally homologous to T7 DNAP‐K536

**Fig. 1:**
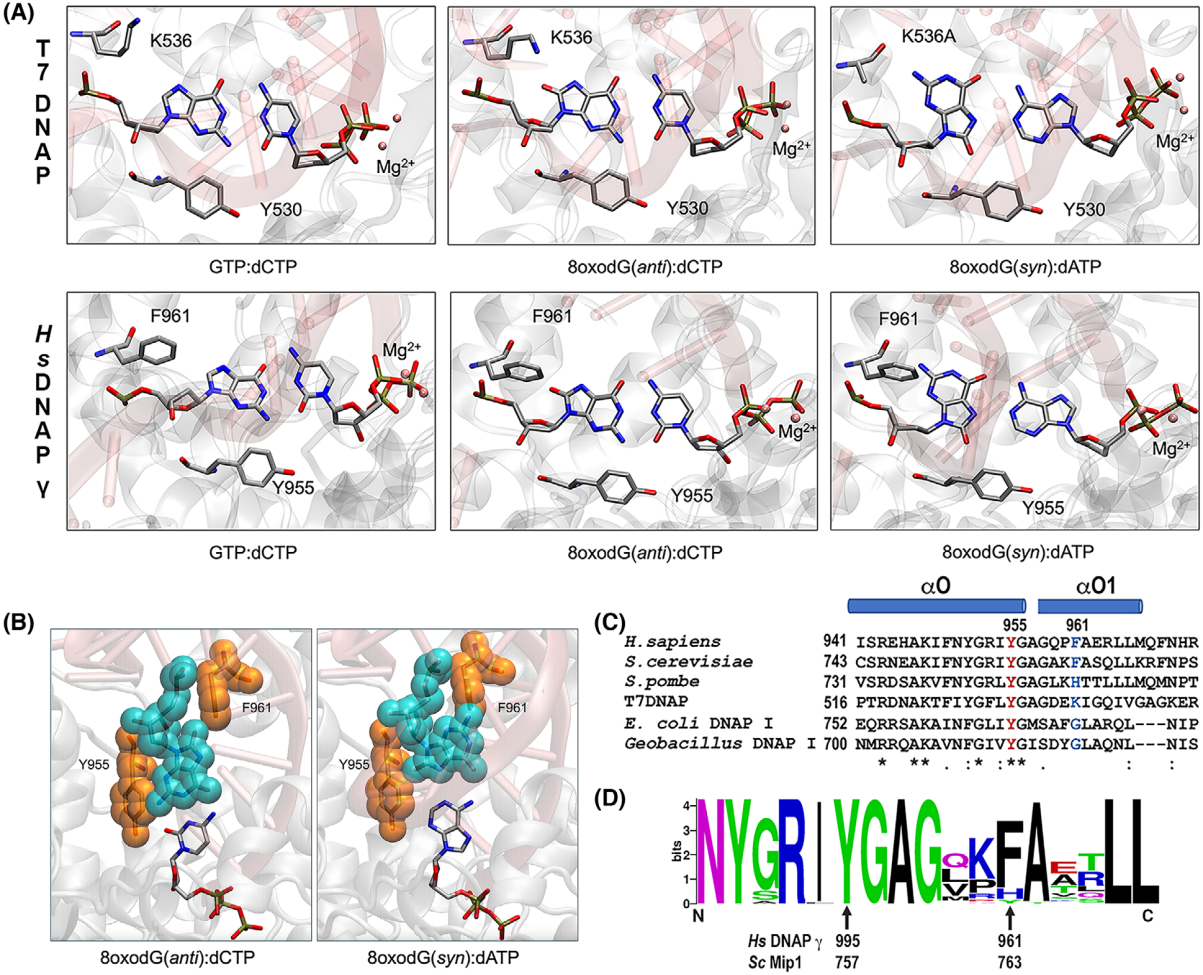
Structural comparison for dCTP (correct) and dATP (mutagenic) incorporation by T7 DNA polymerase and HsPolγ across 8‐oxodG. (A) Ribbon and surface representation of wild‐type T7 DNAP in complex with GTP:dCTP (PDB: 1T8E) and 8‐oxodG:dCTP (PDB: 1TK0) and the T7 DNAP‐K536A mutant 8‐oxodG:dATP (PDB: 1ZYQ) [6, 14]; HsPolγ with GTP:dCTP (PDB: 4ZTZ) [19] and the structural modeling of HsPolγ during dCTP and dATP incorporation across 8‐oxodG. The crystal structures of T7DNAP highlight the interaction between the epsilon amino group of K536 and the O^8^ group of 8‐oxodG, which stabilizes the 8‐oxodG(*anti*):dCTP pair. These crystal structures also underscore the need to remove the large lysine side chain of T7DNAP‐K536 to accommodate the 8‐oxodG(*syn*):dATP pair in the polymerase's active site. The structural modeling of HsPolγ presents a superposition of the 8‐oxodG(*anti*):dCTP and 8‐oxodG(*syn*):dATP pairs in the active site of HsPolγ. This superposition suggests that the 8‐oxodG(*syn*) conformation faces steric clashes, particularly with residue HsPolγ‐F961. Residue HsPolγ‐F961 is located in a position that corresponds to the position of T7DNAP‐K536. Structures were rendered with visual molecular dynamics. (B) Connolly surface representation for dCTP and dATP incorporation by HsPolγ, rendered with visual molecular dynamics, on a modeled 8‐oxodG template. (C) Structural alignment showing helices O and O_1_. The universally conserved Y955 or Y530 in HsPolγ or T7 DNAP respectively is colored in red, whereas the variable residue located six positions after this amino acid is colored in cyan. The corresponding residue in mitochondrial DNA polymerases is a Phe or a His. In the case of *S. cerevisiae* Mip1, the identity of this residue is F763. The multiple sequence alignment was carried out with the software Geneious using the algorithm MUSCLE. The sequences included in the alignment are mitochondrial DNA polymerases from *Homo sapiens* (NCBI ID; KAI2575773.1), *Saccharomyces cerevisiae* (CAD6643831.1), *Schizosaccharomyces pombe* (KAL2313878.1) and T7 DNA Polymerase (UJQ71155) *Escherichia coli* DNA Polymerase I (UJY26831.1) and *G. stearothermophilus* DNA polymerase I (AAB62092.1 D) Sequence logo derived from a sequence alignment which included 51 amino acid sequences from animal and fungal mitochondrial DNA polymerases as described in the material and methods section.

Crystal structures of T7 DNAP in complex with 8‐oxodG(*syn*):dATP and 8‐oxodG(*anti*):dCTP highlight the role of residue K536 in promoting dCMP and limiting dAMP incorporation opposite 8‐oxodG [6, 14]. Residue T7 DNAP‐K536 stabilizes the 8‐oxodG(*anti*) conformation by interacting with the 8‐oxo group and obtruding the 8‐oxodG(*syn*) conformation by nonfavorable steric interactions [6] (Fig. 1A). Attempts to obtain a crystal structure of an 8‐oxodG(*anti*):dCTP pair using wild‐type T7 DNAP resulted in an open conformation of the polymerase, with no bound incoming nucleotide. In contrast, the T7 DNAP‐K536A mutant eliminates these steric clashes, enabling the crystallization of the 8‐oxodG(*syn*):dATP pair [6, 14]. The crystal structure of HsPolγ in complex with a G:dCTP base pair reveals that the HsPolγ‐F961 residue occupies a structurally similar position to T7 DNAP‐K536 [19]. In both DNAPs, residues T7 DNAP‐K536 or HsPolγ‐F961 assemble the upper part of a dNTP binding pocket, whereas a conserved tyrosine residue (T7DNAP‐Y530 or HsPolγ‐Y955) shapes the lower part of the dNTP binding pocket (Fig. 1A). Residue HsPolγ‐Y955 and its structurally homologous Y390 in φ 29 DNA polymerase promote dCMP incorporation opposite the 8‐oxodG lesion by blocking the *syn* conformation of 8‐oxodG [15, 34]. A structural superimposition of HsPolγ with an 8‐oxodG(*anti*):dCTP base pair shows that the 8‐oxodG(*anti*) base could be accommodated into the dNTP binding pocket; however, residue HsPolγ‐F961 is predicted to impinge the 8‐oxodG(*syn*) conformation by creating unfavorable steric interactions. The latter is in a homologous fashion to residue K536 (a residue with a long side chain) in T7DNAP that blocks dATP incorporation via steric interactions that obtrude an 8‐oxodG(*syn*):dATP pair (Fig. 1A). Thus, a superposition of the 8‐oxodG(*anti*):dCTP pair in the active site of HsPolγ shows no significant steric or electrostatic clashes. In contrast, a model of the 8‐oxodG(*syn*):dATP pair reveals that residues HsPolγ‐F961 and HsPolγ‐Y955 are positioned to create steric clashes with the N_1_ and N_2_ atoms, as well as the O^8^ group of 8‐oxodG, thereby hindering dATP incorporation (Fig. 1A,B).

**Fig. 2:**
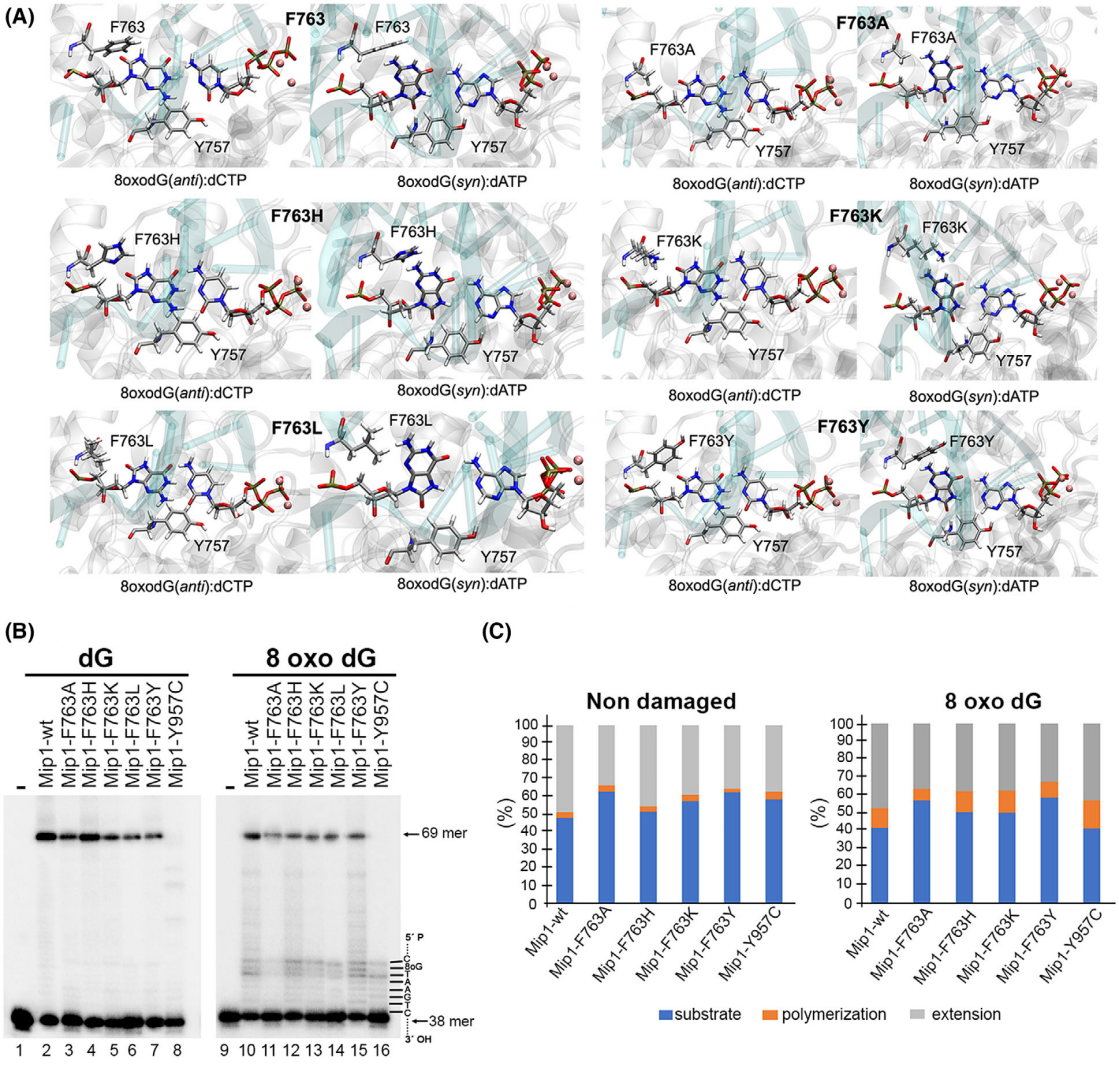
Structure rationale for the use of Mip1‐F763 and Y757 to assemble a steric gate that obtrudes the 8‐oxodG *syn* conformation. (A) Structural models of wild‐type Mip1 and its point mutants at residue 763 rendered with visual molecular dynamics representing the 8‐oxodG(*anti*):dCTP pair for correct incorporation and the 8‐oxodG(*syn*):dATP pair for mismatch incorporation, show significant active site rearrangements. These rearrangements are necessary to accommodate the incorporation of dCTP or dATP opposite 8‐oxodG and involve changes in the orientation of residue 763. (B) Wild‐type Mip1 and point mutants in residue F763 efficiently bypass 8‐oxodG. The left panel shows primer extension of wild‐type and point mutants on non‐damaged DNA (Lane 2–8) and the right panel shows primer extension on an 8‐oxodG damaged DNA (Lane 10–16) by the same enzymes. Lanes 1 and 9 are negative controls (no enzyme in the reaction). (C) Bar Plots indicating the amount of unprocessed substrate (blue), polymerization products before the initial 8‐oxodG or G (orange), extension past the 8‐oxodG or G (gray) as a percentage of the total labeled products/substrate present in the reaction. Experiments were executed by triplicate and a representative reaction is present.

In mitochondrial DNA polymerase from *Saccharomyces cerevisiae* (Mip1), the corresponding amino acids to HsPolγ‐F961 and HsPolγ‐Y955 are F763 and Y757, respectively (Fig. 1C,D). To investigate whether residues Mip1‐F763 and Y757 are responsible for modulating dAMP or dCMP incorporation across 8‐oxodG, we modified those residues using site‐directed mutagenesis. We hypothesized that mutants with a decreased van der Waals radius and without the ability to establish a hydrogen bond with the O^8^ group of 8‐oxodG (F763A and F763L) would facilitate the stabilization of the 8‐oxodG(*syn*) conformation and therefore dAMP incorporation (Fig. 2A). On the other hand, mutants with the potential to establish a hydrogen bond (F763W, F763H, F763K and F763Y) with the O^8^ group of 8‐oxodG would favor the 8‐oxodG(*anti*) conformation and therefore dCMP incorporation (Fig. 2A). We also constructed a Mip1‐Y757C mutant that potentially favors the 8‐oxodG(*syn*) conformation by increasing the size of the dNTP binding pocket, as previously demonstrated for the HsPolγ‐Y955C mutant [15]. In order to predict the effect of the point mutations for dATP or dCTP incorporation opposite 8‐oxodG by Mip1, we measured the Solvent Accessible Surface Area (SASA) for wild‐type Mip1 and each minimized F763 mutant for 8‐oxodG(*syn*):dATP and 8‐oxodG(*anti*):dCTP pairs (Table 1). Among the Mip1 mutants, the F763A mutation exhibited the highest SASA for both dATP and dCTP incorporations, with a greater value for the 8‐oxodG(*syn*) compared with the (*anti*) conformation. As *syn* is the preferred conformation for 8‐oxodG, this increase in surface area could promote the stabilization of the 8‐oxodG(*syn*):dATP pair and therefore dATP incorporation. F763H and F763Y mutations showed SASA values that are similar to wild‐type Mip1. This similar SASA may disrupt the 8‐oxodG(*syn*) conformation, suggesting that bulky histidine and tyrosine amino acids could exhibit behavior more aligned with the wild‐type preference than wild‐type Mip1, thereby preventing the formation of an 8‐oxodG(*syn*):dATP pair. Additionally, these residues could potentially form a hydrogen bond with the O^8^ group of 8‐oxodG(*anti*), stabilizing the 8‐oxodG(*anti*):dCTP pair to facilitate dCTP incorporation.

**Table 1:** Solvent Accessible Surface Area (SASA) analysis for 8‐oxodG(*syn*):dATP, 8‐oxodG(*anti):*dCTP wild‐type and F763 mutants.

	SASA (Å^2^)	
	8‐oxodG(*syn*):dATP	8‐oxodG(*anti*):dCTP
Wild‐type	1883.15	1797.63
F763A	1955.64	1849.40
F763H	1892.04	1803.20
F763K	1952.63	1848.90
F763L	1948.85	1843.35
F763Y	1882.95	1798.94

### Mip1‐F763 mutants are catalytically active and efficiently bypass 8‐oxodG

Mip1 mutants were constructed on an exonuclease/editing‐deficient background that eliminates the two conserved carboxylates in motif I of the Mip1 exonuclease domain (D171A‐E173A). To maximize the detection of all the incorporations occurring opposite 8‐oxodG, we decided to use an exonuclease‐deficient background, although the excision of erroneous dAMP incorporation at 8‐oxodG is not specifically favored by family‐A DNA polymerases [6, 7]. Mip1 mutants, except for Mip1‐F763W, which resulted in low expression yields and were prone to proteolysis, were purified to homogeneity (data not shown). To investigate whether point mutants in residues Mip1‐F763 and Mip1‐Y757 are catalytically active, we first address their primer extension capabilities on canonical and 8‐oxodG templates. To assess lesion bypass opposite 8‐oxodG, we use dNTPs at a concentration of 1 μm, which is considered a low nucleotide concentration in human mitochondria [35]. The template substrate contains an 8‐oxodG lesion after six positions from the 3′‐OH end of the primer strand. As a comparison point, we used an identical primer‐template that harbors deoxyguanosine instead of 8‐oxodG. Nucleotide incorporation on undamaged dsDNA shows that at low dNTP concentrations, wild‐type and F763 mutants readily extend the 38‐mer primer to a 69‐mer product (Fig. 2B,C). In contrast, the Mip1‐Y757C mutant is unable to reach the end of the template, presumably due to a low catalytic efficiency. However, a 10‐fold increase of Mip1‐Y757C in combination with high dNTP concentration generates full‐length products (data not shown). The low catalytic efficiency of Mip1‐Y757C correlates with the low enzymatic activity of its corresponding mutant in HsPolγ (HsPolγ‐Y955C), which decreases the catalytic efficiency for canonical dNTP incorporation by nearly 4000‐fold [15]. To investigate whether 8‐oxodG is a barrier to dNTP incorporation by Mip1, we measured the effect of dNTP incorporation opposite this lesion. When wild‐type and the different Mip1 mutants were tested on the 8‐oxodG lesion‐containing template, distinctive short products appeared when the polymerase approached, incorporated, and extended from 8‐oxodG (Fig. 2B, lanes 10–16). The presence of those products indicates that wild‐type Mip1 and its mutants stall when they encounter an 8‐oxodG lesion, in a similar fashion to HsPolγ and T7 DNAP [6, 35]. However, in comparison with other oxidative lesions such as apurinic sites or thymine glycol, 8‐oxodG exerts a mild blockade of DNA replication [36].

### Mip1‐F763 and Mip1‐Y757C mutants have an altered preference for dAMP incorporation across 8‐oxodG

**Fig. 3:**
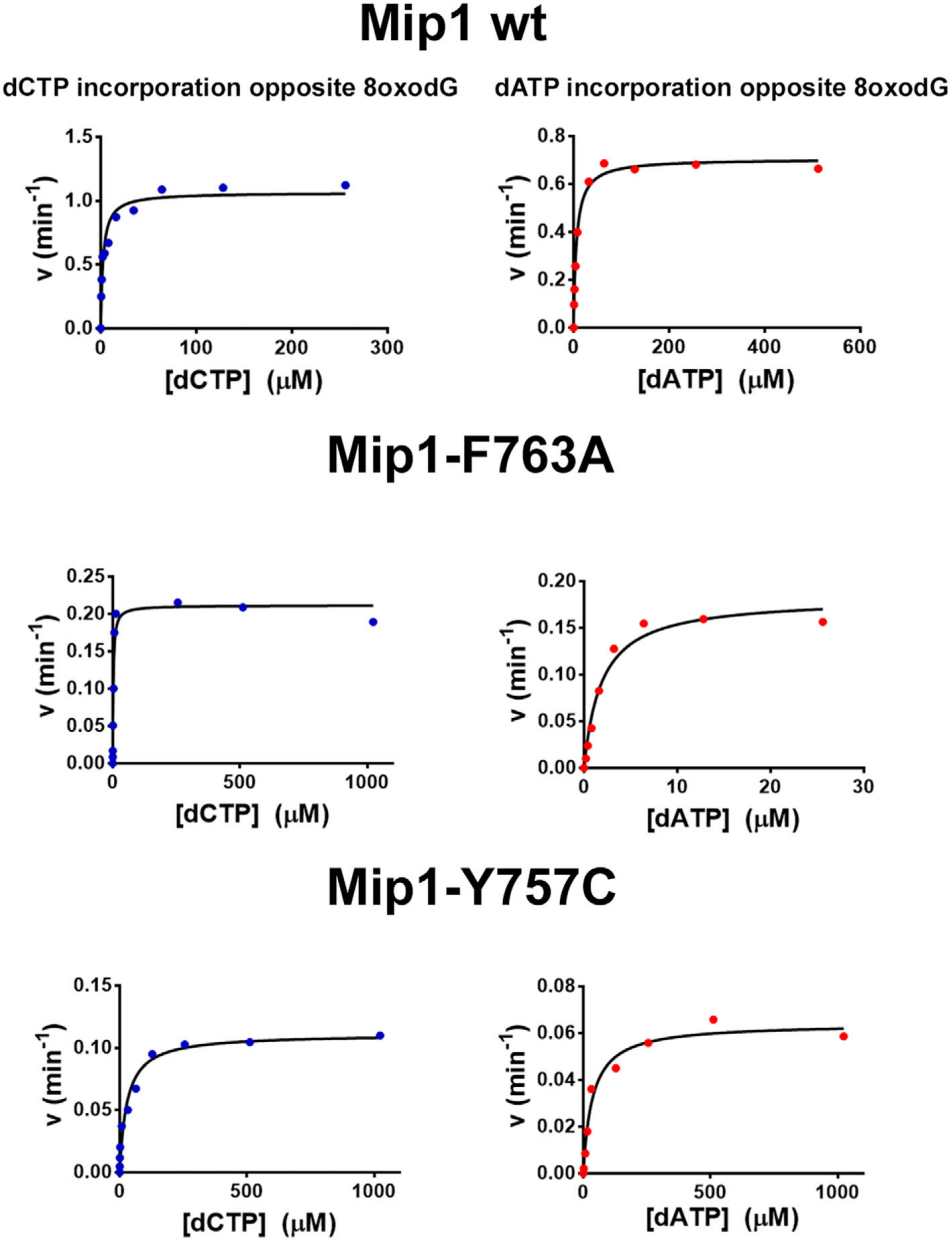
Steady‐state kinetics analysis showing dAMP or dCMP nucleotide incorporation opposite 8‐oxodG by wild‐type Mip1, F763A, and Y757C mutants. 10 nm of DNA‐damaged 8‐oxodG was mixed with 0.5, 2.5, or 4 nm of wild‐type Mip1, Mip1‐F763A, or Mip1‐Y757C, respectively, and was preincubated at 37 °C for 5 min. The reactions were initiated by adding different concentrations of dAMP or dCMP. The value of velocity was plotted against dNTP concentration. The reactions were conducted three times, and the mean of Vmax and Km is reported.

We estimated the preference of dNTP incorporation on 8‐oxodG by measuring the kinetic parameters for correct and incorrect incorporation under a steady‐state approach [37]. To investigate whether mutations in Mip1 yield an altered preference for dATP or dCTP incorporation opposite 8‐oxodG, we performed several steady‐state kinetics assays using a radiolabeled 19‐mer primer annealed to a 28‐mer primer‐template; in this template, the 8‐oxodG lesion is the first base to be used as a substrate. Using this substrate, we determined the Michaelis–Menten constants for wild‐type and Mip1 mutants (F763A, F763H, 763K, F763L, F763Y, and Y757C) (Fig. 3 and Table 2). We first measured the catalytic parameters of wild‐type Mip1 for dAMP and dCMP incorporation opposite an undamaged template (deoxyguanosine). Steady‐state kinetic parameters by wild‐type Mip1 for dAMP and dCMP incorporation on a deoxyguanosine template indicate that this enzyme incorporates dCMP with an efficiency of 99.95% (Table 2). The discrimination factor of nearly 2000 for correct and incorrect nucleotides across an undamaged template is like the values observed for other high‐fidelity DNAPs [15, 22]. A mutant Mip1‐Y757C retains its ability to incorporate dCMP more efficiently than dAMP, although its enzymatic efficiency decreases ~ 1400‐fold in comparison to wild‐type Mip1, and this point mutant shows a 30‐fold increase for dAMP misincorporation (Table 2). The lower catalytic efficiency and the decrease in nucleotide incorporation fidelity are expected, as residues homologous to Mip1‐Y757 are involved in nucleotide incorporation and fidelity in other high‐fidelity DNAPs [15, 38, 39, 40, 41]. Furthermore, yeast harboring a Mip1‐Y757C pathogenic variant is unable to grow on nonfermentable carbon sources, supporting the essential role of this residue in assembling the polymerase's active site [42]. Next, we measured the preference to incorporate dATP *versus* dCTP in an 8‐oxodG template. Nucleotide selectivity was estimated using the misincorporation frequency (*f*_misincorp_) = (*K*_cat_/*K*_M_)_dATP_/ (*K*_cat_/*K*_M_)_dCTP_ to obtain the percentage of dATP and dCTP incorporation (Table 2) [8, 43]. Mip1 preferentially incorporates dCMP based on a 2.5 lower *K*_M_ for dCMP incorporation and nearly twofold larger *K*_cat_ in comparison with dAMP incorporation (Table 2). Mip1 incorporates dCMP in 85% of its incorporation events across an 8‐oxodG template. This value contrasts with HsPolγ, which incorporates dCMP opposite 8‐oxodG, with a discrimination factor (*f*_ins_) of 2500 compared to dAMP incorporation [15]. However, Mip1 presents similar dCMP incorporation values compared to T7 DNAP and Polγ from *Xenopus laevis*, which are 63% and 73%, respectively [43, 44]. Mutations in Mip1‐F763 alter the incorporation ratio of dAMP *versus* dCMP opposite 8‐oxodG. For instance, Mip1‐F763A and Mip1‐F763K mutants decreased their incorporation fidelity on 8‐oxodG, as they misincorporate dAMP with nearly similar efficiency to dCMP (Table 2 and Fig. 3). Mip1‐F763H, Mip1‐F763L, and Mip1‐F763Y mutants misincorporate dATP in 20.4%, 26.3%, and 26% of their incorporation events, values that are higher than the 15% dAMP incorporation present by wild‐type Mip1 (Table 2). Surprisingly, all point mutants increase dATP misincorporation across 8‐oxodG. Our data agree with the hypothesis that a substitution of the nonconserved phenylalanine (F757) to alanine amino acid increases the volume of the catalytic pocket and promotes the formation of an 8‐oxodG(s*yn*):dATP pair in the polymerase active site. Unexpectedly, Mip1‐F763K, Mip1‐F763H, and Mip1‐F763Y mutants that have the potential to establish a hydrogen bond with 8‐oxodG(*anti*) also decrease dCTP incorporation. Our data suggest that the imidazole, hydroxyl, and epsilon amino groups of Mip1‐F763H, Mip1‐F763Y, and Mip1‐F763K respectively, are not able to form H‐bonds with the O^8^ group of 8‐oxodG in its *anti*conformation. An amino acid sequence analysis including animal and fungi mitochondrial DNAPs shows that phenylalanine is present at the corresponding position to Mip1‐F763 in 86% of the selected sequences, indicating a strong preference for the conservation of this residue in mitochondrial DNA polymerases. A Mip1‐Y757C mutant decreases dCTP incorporation from 85 % to 68% (Table 2) according to the role of the conserved tyrosine residue for assembling the lower segment of the dNTP binding pocket and hampering the *syn* conformation of 8‐oxodG, as demonstrated by Copeland and coworkers [15].

**Table 2:** Steady‐state kinetic parameter for the incorporation of dATP and dCTP on G or 8‐oxodG templates by mutants and Mip1 wild type.

DNA polymerase	Template	Incoming nucleotide	*K*_M_ (μm)	*k*_cat_ (min^−1^)	*k*_cat_/*K*_M_	*f*_ins_ ^a^	% incoming dAMP^b^	% incoming dCMP
Wild‐type exo‐	G	dATP	2.14 ± 0.16	0.228 ± 0.004	0.107 ± 0.026	1915	0.0518	
	G	dCTP	0.0167 ± 0.0026	3.44 ± 0.052	205.85 ± 20			99.948
Y757C exo‐	G	dATP	25.5 ± 3.11	0.0549 ± 0.003	0.00215 ± 0.00096	71	1.48	
	G	dCTP	1.289 ± 0.069	0.185 ± 0.0055	0.143 ± 0.08			98.52
Wild‐type exo‐	8‐oxodG	dATP	6.3 ± 0.41	0.94 ± 0.009	1.5 × 10^−1^ ± 0.022	5.6	14.9	
	8‐oxodG	dCTP	2.5 ± 0.33	2.13 ± 0.027	8.5 × 10^−1^ ± 0.081			85.1
F763A exo‐	8‐oxodG	dATP	1.884 ± 0.19	0.0609 ± 0.005	3.2 × 10^−2^ ± 0.026	1.15	46.5	
	8‐oxodG	dCTP	2.29 ± 0.28	8.45 × 10^−2^ ± 0.004	3.7 × 10^−2^ ± 0.014			53.5
F763H exo‐	8‐oxodG	dATP	5.2 ± 0.71	1.8 × 10^−1^ ± 0.009	3.5 × 10^−2^ ± 0.013	3.9	20.4	
	8‐oxodG	dCTP	2.4 ± 0.27	3.3 × 10^−1^ ± 0.009	1.37 × 10^−1^ ± 0.033			79.6
F763K exo‐	8‐oxodG	dATP	2.3 ± 0.19	1.3 × 10^−2^ ± 0.0008	5.6 × 10^−3^ ± 0.004	1.2	45.4	
	8‐oxodG	dCTP	2.1 ± 0.38	1.4 × 10^−2^ ± 0.002	6.7 × 10^−3^ ± 0.005			54.6
F763L exo‐	8‐oxodG	dATP	7.6 ± 0.35	6.1 × 10^−2^ ± 0.0017	8.0 × 10^−3^ ± 0.0049	2.75	26.3	
	8‐oxodG	dCTP	4.1 ± 0.34	9.0 × 10^−2^ ± 0.0025	2.2 × 10^−2^ ± 0.007			73.7
F763Y exo‐	8‐oxodG	dATP	7.2 ± 0.69	3.2 × 10^−1^ ± 0.009	4.4 × 10^−2^ ± 0.013	2.84	26	
	8‐oxodG	dCTP	3.6 ± 0.31	4.5 × 10^−1^ ± 0.011	1.25 × 10^−1^ ± 0.035			74
Y757C exo‐	8‐oxodG	dATP	34.0 ± 3.76	1.6 × 10^−2^ ± 0.0015	4.7 × 10^−4^ ± 0.0004	2.12	32	
	8‐oxodG	dCTP	27.1 ± 4.35	2.8 × 10^−2^ ± 0.0036	1 × 10^−3^ ± 0.0008			68

^a^ *f*_ins_, Value of (*k*_cat_/*K*_M_)_dCTP_/(*k*_cat_/*K*_M_)_dATP_ for each mutant and wild type.

^b^ % incoming dAMP, value is defined by the equation (*f*_misinsertion_) = (*k*_cat_/*K*_m_)dATP/(*k*_cat_/*K*_m_)dCTP % misincorporation = (*f*/[1 + *f*]) * 100.

### Mip1‐F763 mutants affect extension past an 8‐oxodG lesion

**Fig. 4:**
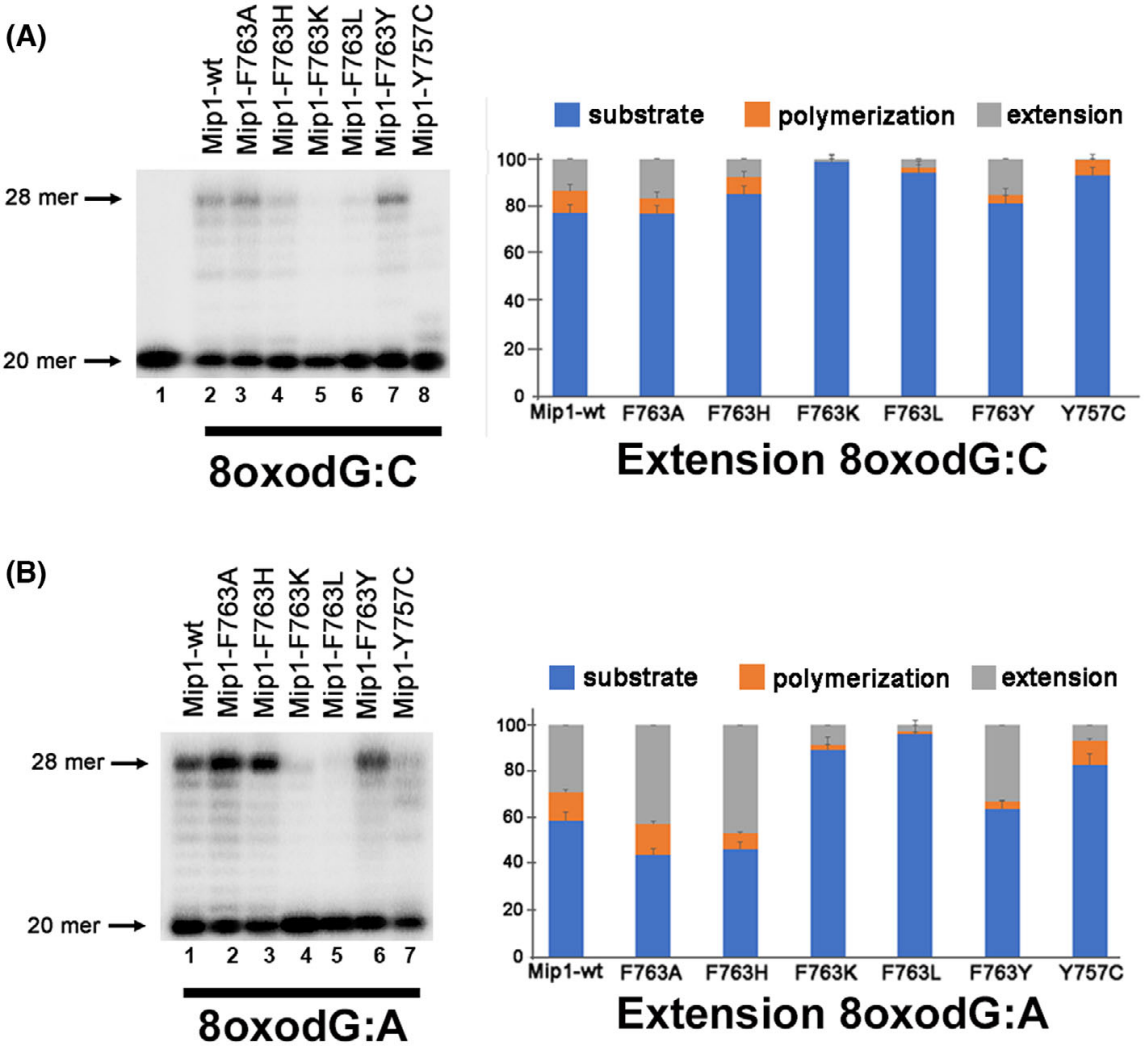
Primer extension by wild‐type Mip1 and point mutants F763 and Y757C using an 8‐oxodG:C or an 8‐oxodG:A substrate at low nucleotide concentrations. The reactions were run on 17% denaturing polyacrylamide gels, illustrating primer extension after (A) 8‐oxodG:C and (B) 8‐oxodG:A incorporation, along with the corresponding quantification. The quantification represents the percentage of unprocessed substrate (blue), polymerization (orange), and extension (gray). The standard deviation of triplicate experiments is represented by error bars. Reactions contained 10 nm of DNA substrate and 20 nm of DNA polymerase, which were preincubated at 37 °C for 5 min before initiating the reaction with 1 μm of each of the four dNTPs.

In family‐A DNA polymerases, the Hoogsteen base pair of an incorporated 8‐oxodG:A is not recognized as a mismatch and is readily used as a substrate [6, 7, 18]. In order to investigate whether mutations in residue Mip1‐F763 affect the expected ability of Mip1 to elongate from an 8‐oxodG:A (a mismatch) more efficiently than from an 8‐oxodG:C pair (Watson‐Crick) [6, 8, 43], we measured the primer extension capabilities on 8‐oxodG templates paired with dAMP or dCMP (Fig. 4). Like all family‐A DNA polymerases, wild‐type Mip1 presents a greater percentage of polymerization and extension products from an 8‐oxodG:A pair than from an 8‐oxodG:C pair (Fig. 4A lane 2 and 4B lane 1). This phenomenon is also evidenced in the Mip1‐Y757C mutant that synthesizes full‐length elongation products only from the 8‐oxodG:A pair (Fig. 4A lane 8 and 4B lane 7). Although Mip1 bypasses a paired 8‐oxodG lesion, we found a difference in the ability of the F763 mutants during the bypass. Mip1‐F763K and Mip1‐F763L mutants showed a severe defect in elongating after 8‐oxodG:A and 8‐oxodG:C pairs (Fig. 4A lanes 5, 6, and 4B, lanes 4, 5). It is plausible that the linear and flexible side chains of lysine and leucine interfere with the positioning of the templating base after an 8‐oxodG, exacerbating the intrinsic disorder caused by 8‐oxodG at the template base.

**Fig. 5:**
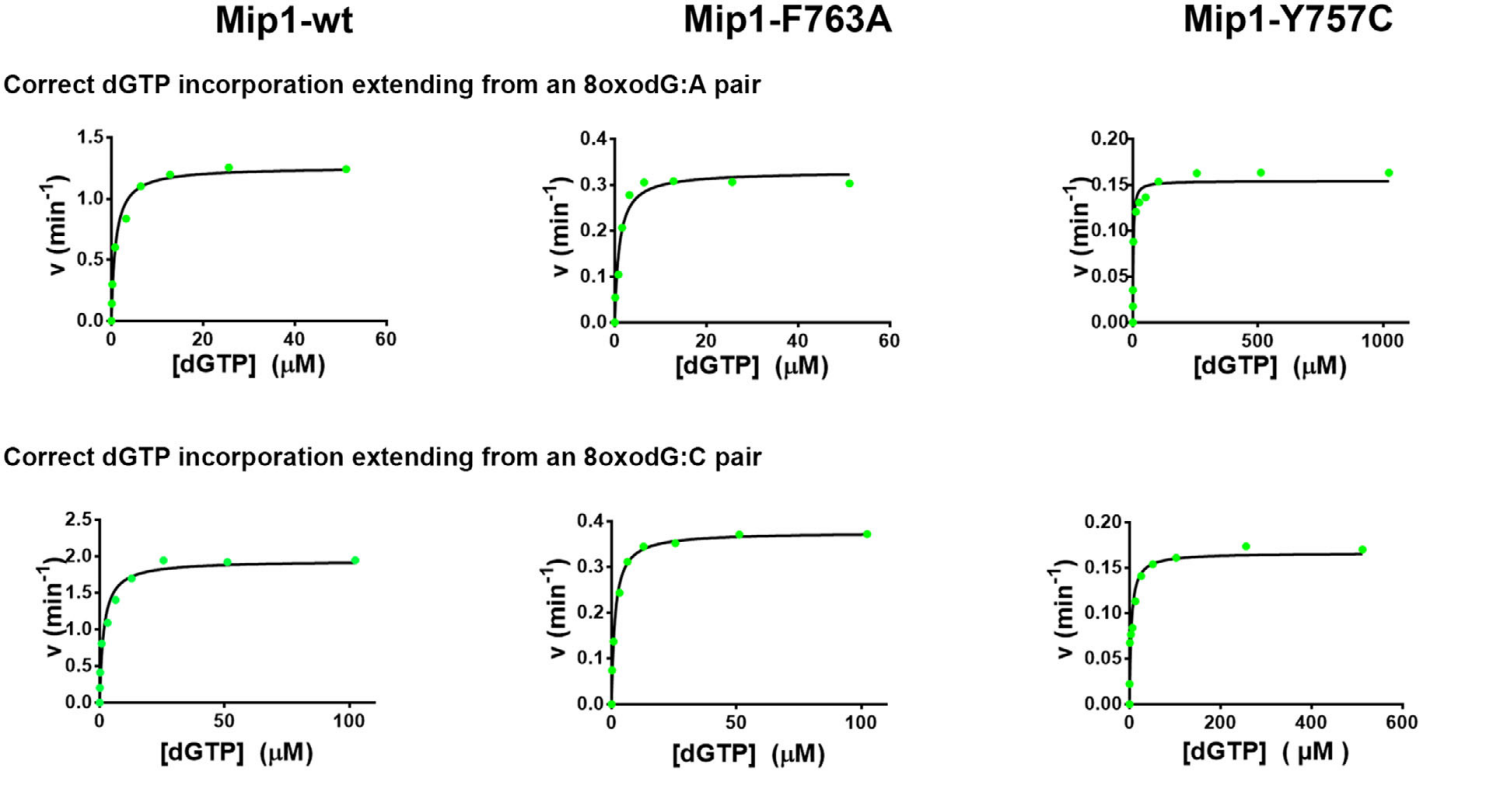
Steady‐state kinetic analysis of correct dGTP incorporation extending from 8‐oxodG:A or 8‐oxodG:C pairs. 10 nm of DNA‐damaged 8‐oxodG were mixed with 0.5, 2.5, or 4 nm of wild‐type Mip1, Mip1‐F763A, or Mip1‐Y757C, respectively. The reactions were preincubated at 37 °C for 5 min and initiated with different nucleotide concentrations as indicated in this figure. The value of velocity was plotted against dNTP concentration. The reactions were conducted three times, and the mean of Vmax and Km is reported.

The rationale for extending from a mismatch more efficiently than from an 8‐oxodG:C base pair is that the 8‐oxodG:A mismatch mimics a T:A canonical base and because the 8‐oxodG:C pair alters the DNA backbone [6, 7]. To study nucleotide incorporation past an 8‐oxodG:A mismatch and an 8‐oxodG:C base pair by wild‐type Mip1 and Mip1‐F763A, we used a 20‐mer primer containing an A or C paired with an 8‐oxodG base in its 3′‐OH end. When we performed a control experiment extending a single dNTP from a G:A mismatch or a G:C base pair, exonuclease‐deficient Mip1 presented a discrimination factor of approximately 2000 (Table 3), indicating that Mip1 is not able to extend from mismatches. In contrast, exonuclease‐deficient Mip1 and the Mip1‐F763A mutant preferentially execute dNTP incorporation from an 8‐oxodG:A mismatch rather than from an 8‐oxodG:C pair (Table 3 and Fig. 5). Both enzymes displayed a *fin* for incorporation past an 8‐oxodG with a value near 1 indicating the similarities for primer extension of an 8‐oxodG:A mismatch or an 8‐oxodG:C base pair (Table 3).

**Table 3:** Steady‐state kinetic parameters to extend 8‐oxodG‐containing base pairs.

DNA polymerase	Template	Incoming nucleotide	*K*_M_ (μm)	*k*_cat_ (min^−1^)	*k*_cat_/*K*_M_	*Fins*
Wild‐type	5‐C 3‐G C‐5	dGTP	0.0158 ± 0.003	3.11 ± 0.06	196.84 ± 20	2029
	5‐C 3‐G C‐5	dGTP	15.13 ± 1.54	1.47 ± 0.021	0.097 ± 0.0136	
	5‐C 3‐8oG C‐5	dGTP	1.68 ± 0.19	3.91 ± 0.037	2.33 ± 0.19	0.879
	5‐A 3‐8oG C‐5	dGTP	0.95 ± 0.089	2.52 ± 0.021	2.65 ± 0.24	
F763A	5‐A 3‐8oG C‐5	dGTP	0.21 ± 0.014	0.16 ± 0.0012	0.76 ± 0.086	304
	5‐A 3‐G C‐5	dGTP	21.3 ± 2.6	0.054 ± 0.0006	0.0025 ± 0.00023	
	5‐C 3‐8oG C‐5	dGTP	1.4 ± 0.098	0.125 ± 0.0009	0.089 ± 0.0092	0.809
	5‐A 3‐8oG C‐5	dGTP	1.00 ± 0.127	0.11 ± 0.008	0.11 ± 0.063	
Y757C	5‐C 3‐G C‐5	dGTP	2.37 ± 0.35	0.36 ± 0.019	0.152 ± 0.054	177.77
	5‐A 3‐G C‐5	dGTP	61.97 ± 4.8	0.053 ± 0.0018	0.000855 ± 0.000038	
	5‐C 3‐8oG C‐5	dGTP	4.0 ± 0.63	0.083 ± 0.0048	0.021 ± 0.0076	1.05
	5‐A 3‐8oG C‐5	dGTP	1.96 ± 0.25	0.039 ± 0.0024	0.020 ± 0.0096	

### Point mutants in residue Phe763 increase the frequency of *petite* mutants *in vivo* without affecting the mtDNA levels

Yeast is a useful model organism to measure the effect of mutations in mitochondrial DNA (mtDNA), and therefore suitable to evaluate the fidelity and efficiency of Mip1 replication in the cellular context: this system has been widely used to better understand pathogenic human DNAP γ mutations [28, 29, 42, 45]. The ability of Mip1, the sole replicative DNAP in yeast mitochondria, to maintain the mtDNA in the cell population can be evaluated by measuring the frequency of respiratory deficient yeast cells harboring large deletions in mtDNA (*rho*ˉ) or completely devoid of mtDNA (*rho* ^0^) [46], which are overall called *petite* cells. Although *petite* cells arise spontaneously at high frequency (> 1%) also in wild‐type strains, if the Mip1 activity is impaired, the *petite* frequency increases. Thus, the frequency of *petite* cells is a measure of mtDNA instability and directly correlates with defects in the synthesis of mtDNA by low fidelity or an overall reduction in replication rates.

**Fig. 6:**
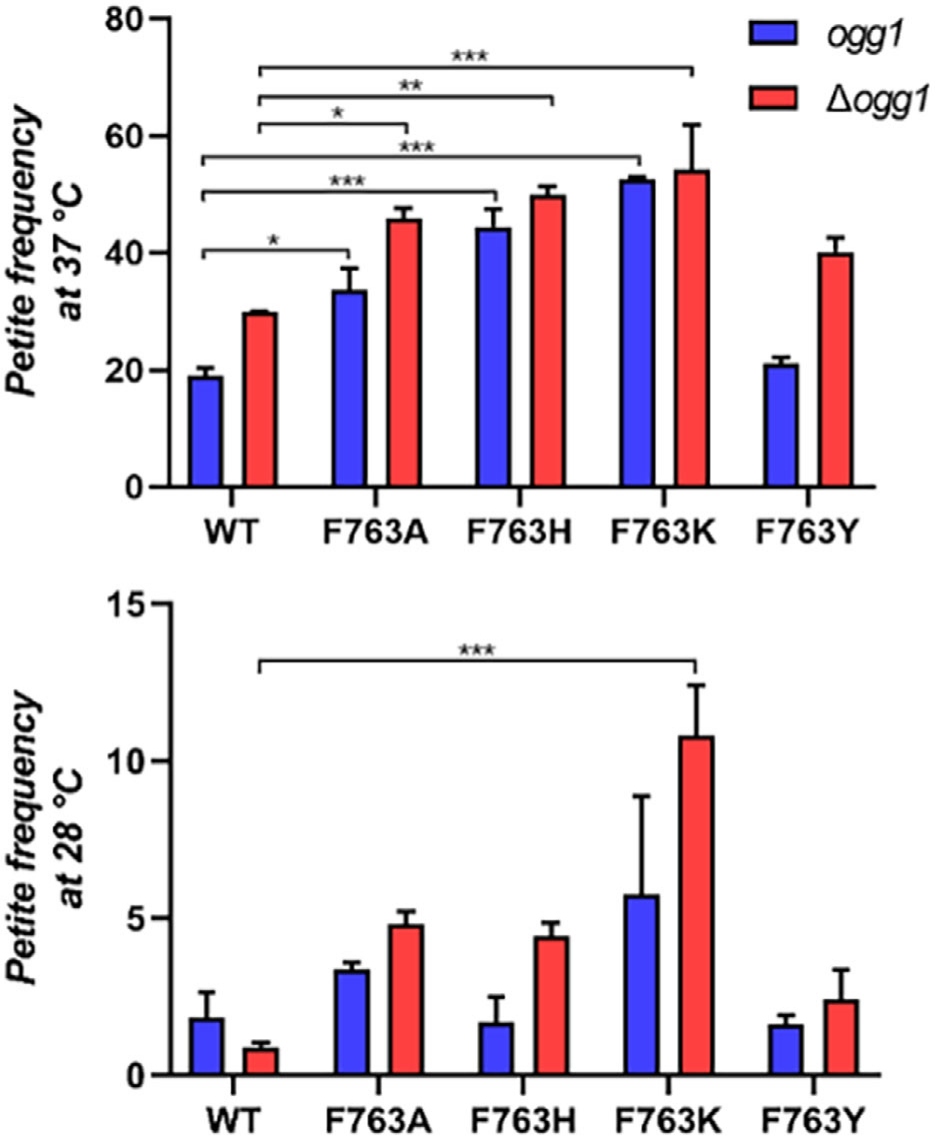
*Petite* mutant frequency measured at 37 and 28 °C in *OGG1*‐proficient (*ogg1*) and ‐deficient (*Δogg1*) yeast strains harboring *MIP1* wild‐type and F763 mutants. The data are reported as mean ± SD. Significant differences (*, *P* < 0.05; **, *P* < 0.01; and ***P < 0.001) were estimated using ANOVA followed by a Bonferroni's *post hoc* test, comparing each mutant with the wild‐type control from each *OGG1* background. The data was obtained from two different colonies evaluated by quadruplicates.

An increase in dAMP incorporation opposite 8‐oxodG and a decrease in the correction of the 8‐oxodG:A mismatch by the Mip1 proofreading mechanism could result in an increase of mtDNA instability, so we decided to measure the formation of *petite* colonies in strain harboring mutations in *MIP1*, either without further mutations or in combination with the deletion of *ogg1*. The latter gene encodes for the 8‐oxodeoxyguanine DNA glycosylase, which removes and mediates the full repair of 8‐oxodG in an 8‐oxodG:C base pair via the Base Excision Repair (BER) pathway [47, 48]. Thus, deletion of *OGG1* results in 8‐oxodG accumulation and in the increase of the *petite* frequency. As shown in Fig. 6, a strain expressing wild‐type Mip1 displayed a *petite* frequency of 1.8% and 18.9% at 28 and 37 °C, respectively. These results are consistent with previous studies [45, 46, 49]. Every point mutation evaluated at the residue F763 displayed a significantly augmented *petite* frequency, at least at 37 °C, with mutations F763H and F763K being the more compromised Mip1 mutants with a 2.3‐ and 2.8‐fold increase at 37 °C (*P* < 0.001), respectively. Interestingly, the mutant F763Y, which conserves the bulky side chain, exhibited no significant difference in comparison with the wild‐type at both 28 and 37 °C. Indeed, it had a lower *petite* frequency at 28 °C and only a 10% increase at 37 °C in comparison with the strain expressing the wild‐type Mip1. The origin of this small difference remains unclear. The F763Y substitution may promote the extension of an 8‐oxodG:C pair or that, in this specific mutant, the 8‐oxodG:A pair is identified as a noncanonical Watson–Crick pair.

The determination of *petite* frequency in an *ogg1Δ* strain expressing wild‐type Mip1 showed a 1.5‐fold increase in comparison with the *OGG1*‐proficient strain at 37 °C, which is in agreement with a previous report [48]. However, this was not the case for the data obtained at 28 °C, where wild‐type Mip1 activity resulted in a lower *petite* frequency in the *OGG1*‐deficient background. Additionally, the deletion of *NTG1*, encoding another DNA glycosylase also involved in the repair of oxidatively damaged pyrimidines, similarly reduced the *petite* frequency at 28 °C (1.8 ± 0.8 and 0.72 ± 0.18 for *NTG1*‐proficient and ‐deficient strains, respectively). We acknowledge that these data might seem counterintuitive; however, the accumulation of 8‐oxodG can induce replicative stress under oxidative stress, a situation that is alleviated by the absence of *OGG1* [50, 51]. Thus, the ability to replicate mtDNA by Mip1 faithfully and efficiently might be slightly better in the absence of these DNA glycosylases, at least at the optimal growth temperature of 28 °C. On the contrary, the deletion of *OGG1* resulted in a further increase in the *petite* frequency in all the Mip1 mutants in residue F763 already at 28 °C. Nonetheless, regardless of the augmented *petite* frequency in the *OGG1‐*deficient strain, the results recapitulated those observed in the *OGG1*‐proficient yeast strain, where only F763Y Mip1 is able to maintain replication capabilities similar to Mip1 wild‐type. Hence, these results, along with the kinetic results described above, support the notion that F763, through the formation of a steric gate, favors the efficient and faithful 8‐oxodG bypass in mitochondrial DNA polymerases.

**Fig. 7:**
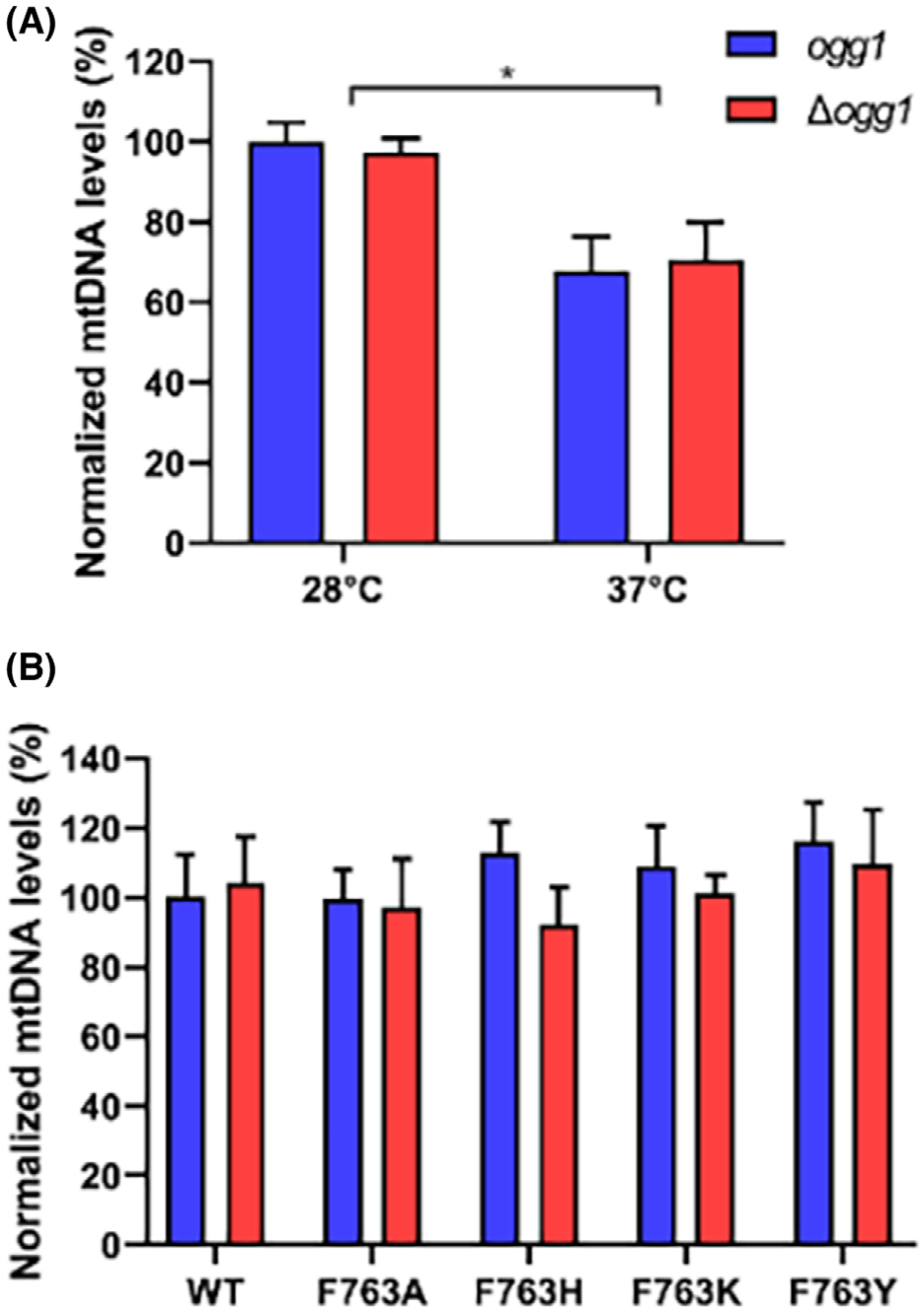
mtDNA levels measured in an *OGG1*‐proficient (*ogg1*) and ‐deficient (*Δogg1*) yeast strains harboring *MIP1* wild‐type and F763 mutants. (A) mtDNA levels in *MIP1* wild‐type strains with or without *OGG1* after growth at 28 or 37 °C. The data are reported as mean ± SD. Significant differences (*, *P* < 0.05) estimated using ANOVA followed by a Bonferroni's *post hoc* test. (B) mtDNA levels in *mip1* mutant strains with or without *OGG1* after growth at 37 °C. The data are reported as mean ± SD and derived from experiments on three different clones and growth conditions.

An increase in the *petite* frequency could also be due to a reduction in the mtDNA levels due to the impairment of the replicative activity of Mip1. By measuring through qPCR the steady‐state levels of mtDNA, we observed that the mtDNA levels were 20% lower when the cells were grown at 37 °C compared to 28 °C (Fig. 7A). However, at 37 °C, the temperature at which a greater increase in the *petite* frequency was observed for some mutants, the mtDNA levels were similar for all *mip1* mutant strains, with or without *OGG1*, suggesting that the difference in *petite* frequency cannot be ascribed to altered mtDNA levels (Fig. 7B).

### Point mutants in residue F763 affect Ery^R^ mutant frequency

**Fig. 8:**
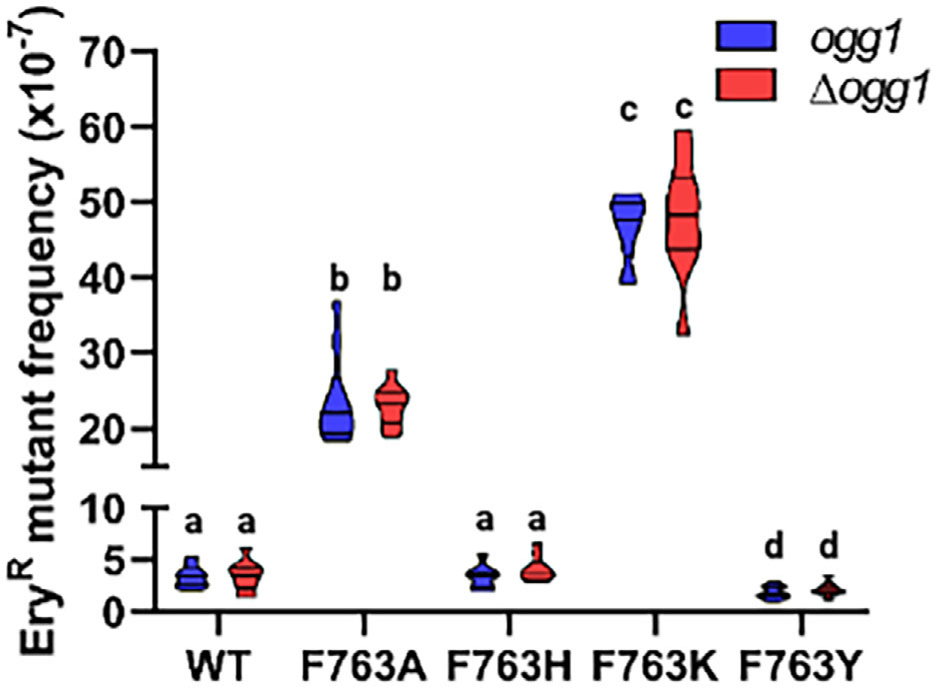
Ery^R^ mutant frequency measured in an *OGG1*‐proficient (*ogg1*) and ‐deficient (*Δogg1*) yeast strains harboring *MIP1* wild‐type and F763 mutants. For each strain, Ery^R^ frequencies of 10 single colonies are reported as median in violin plot. Different letters denote significant differences using a Kruskal–Wallis test followed by a *post hoc* Dunn's test with *P* < 0.05. Mutants with same letters represent do not bear statistical difference. Different lowercase letters in the graph indicates statistical differences between treatments. The statistical difference is not significant with same lowercase letter.

Changes in dAMP incorporation opposite 8‐oxodG and in the correction of the 8‐oxodG:A mismatch could change the *in vivo* fidelity of replication. In order to determine the fidelity of replication, we measured the frequency of spontaneous mutants that become resistant to erythromycin. In yeast, resistance to erythromycin is acquired through specific point mutations in the mtDNA gene encoding the 21S rRNA. Deletion of *OGG1* in the presence of wild‐type Mip1 did not affect the Ery^R^ mutant frequency, as previously reported [48]. Mutations at Position 763 showed different effects, being F763H neutral, F763A and F763K mutagenic, resulting in a 7‐ and a 14‐fold increase in the Ery^R^ point mutability, respectively, and F763Y antimutagenic, resulting in a twofold decrease (Fig. 8). Also, in strains harboring Mip1 variants, the lack of *OGG1* did not affect the mtDNA point mutability (Fig. 8). Comparing these results with the values of the *petite* frequency, there is no correlation between mtDNA point mutability and mtDNA extended mutability/instability.

Mip1 misincorporates dCTP correctly in approximately 15% of its incorporation events (Table 2). Mip1‐F763A and Mip1‐F763K mutants decreased their incorporation fidelity on 8‐oxodG, as they misincorporate dAMP in nearly 50% of the events (Table 2). This correlates with their sevenfold and 14‐fold increase in the Ery^R^ point mutability, respectively. The slight antimutagenic effect observed with the F763Y mutant, as measured by Ery^R^, is unexpected as this mutant misincorporates dATP in 26% of its events. We hypothesize that, unlike the wild‐type Mip1, this mutant might recognize the 8‐oxodG:A pair as a mismatch, or it may show an overall increase in fidelity when incorporating canonical dNTPs. The Mip1‐F763H, Mip1‐F763L, and Mip1‐F763Y mutants misincorporate dAMP in 20.4%, 26.3%, and 26% of their incorporation events, respectively, which is higher than the 15% dATP misincorporation seen with wild‐type Mip1 (Table 2).

## Discussion

The *anti* or *syn* conformation that adopts 8‐oxodG, which templates for faithful or incorrect dNTP incorporation, determines its miscoding potential [6, 7, 12, 52]. Mitochondria are the primary ROS source generated by superoxide production as a by‐product of mitochondrial respiration, and it is estimated that 8‐oxodG can be present in nearly 1% of all deoxyguanosines in mammalian mitochondria [53, 54, 55]. Thus, it is expected that mitochondrial DNA polymerases have evolved mechanisms to preferentially promote dCTP incorporation on an 8‐oxodG template. A common mechanism to do so is the stabilization of the 8‐oxodG(*anti*) conformation before nucleotide incorporation. T7 DNA polymerase and DNA Polι promote faithful dCTP incorporation by establishing hydrogen bond interactions with the O^8^ atom of the 8‐oxodG lesion in its *anti*conformation via lysine and threonine residues, respectively [6, 11]. Yeast and animal mitochondrial DNA polymerases harbor a bulky phenylalanine incapable of stabilizing via hydrogen bond interactions in 87% of the sequences analyzed, except for a few homologs that harbor histidine or tyrosine. Although the marked conservation of a Phe instead of a His or Tyr, which could stabilize the 8‐oxodG(*anti*) conformation, mitochondrial DNA polymerases are extremely efficient and flawless in comparison to other DNA polymerases. In the case of dCTP for incorporation across 8‐oxodG, yeast Mip1 correctly incorporates dCMP in 85% of its events (Table 2), a value that is similar to the 90% of correct dCMP incorporation events by HsPolγ [56].

In this work, we hypothesized that we could enhance dCMP incorporation opposite 8‐oxodG by introducing amino acids able to interact with the 8‐oxodG(*anti*) conformation and at the same time induce a snug/narrow polymerase active site. We designed Mip1 mutants F763A and F763L aimed at increasing dAMP incorporation. All Mip1 mutants, except for F763Y, increase the SASA of the 8‐oxodG(*syn*):dATP pair from 72 to 9 Å^2^. Specifically, the F763A mutant leads to a substantial increase in SASA, greater than 70 Å^2^, suggesting that the 8‐oxodG(*syn*):dATP pair could be more easily accommodated in the active site. In the same token, the F763L mutant decreases the SASA by 66 Å^2^, indicating that this mutation may also favor the 8‐oxodG(*syn*):dATP pair, though to a lesser extent than the F763A mutant (Table 1). According to our predictions, the Mip1 mutants F763A and F763L decrease faithful dCMP incorporation from 85% to 53% and 73%, respectively, while increasing dATP misincorporation to 47% and 27%, respectively. A similar mutation in T7 DNA polymerase (K536A) allows the crystallization of an 8‐oxodG(*syn*):dATP complex in the polymerase active site and mainly incorporates dATP at an 8‐oxodG lesion [14]. Surprisingly, the F763H, F763Y, and F763K mutants, despite having the potential to form hydrogen bonds with 8‐oxodG and favor the 8‐oxodG(*anti*):dCTP base pair, reduce dCMP incorporation to 79%, 74%, and 54%, respectively, while promoting dATP misincorporation. The F763H and F763K mutants decrease the SASA (Table 1), leading us to hypothesize that these mutants may not be able to effectively interact with the O^8^ group of the 8‐oxodG lesion. The F763Y mutant also increases dATP incorporation from 15% to 26% compared to wild‐type Mip1. Structural modeling suggests that the side chain of this residue does not undergo significant rearrangement, indicating that the active site accommodates the 8‐oxodG lesion similarly to the wild‐type enzyme. Interestingly, the hydroxyl group of the F763Y mutant is not positioned to form a hydrogen bond with the O^8^ group of 8‐oxodG, suggesting that this mutant may not preferentially incorporate dCTP at an 8‐oxodG template.

8‐oxodG can stall replicative DNA polymerases, but unlike apurinic sites or thymine glycol, it does not cause a strong block for most DNA polymerases, including human DNA polymerase γ [35, 57, 58]. We observed differences in the ability of F763 mutants to bypass an 8‐oxodG lesion. Specifically, the Mip1‐F763K and Mip1‐F763L mutants exhibit a severe defect in elongation when encountering 8‐oxodG paired with either A or C. This correlates with nucleotide incorporation by T7 DNA polymerase that reduces primer extension efficiency by 300‐fold using 8‐oxodG:C pairs and 50‐fold for an 8‐oxodG:A pair [43]. The increased inhibition for 8‐oxodG:C pairs is thought to result from structural changes in the sugar‐phosphate backbone and subtle alterations in the sugar component of the templating nucleotide [6]. Although structural studies suggest that the 8‐oxodG:A pair does not significantly affect the geometry of base pairing at the active site, this pair may still cause misalignment of either the primer or the templated base [6].

In sum, our results suggest that the active site of mitochondrial DNA polymerases evolved to harbor a bulky amino acid as a central component of a steric gate that modulates dCMP incorporation at 8‐oxodG by occluding the formation of an 8‐oxodG(*syn*):dATP base pair. This observation correlates with the increase in the number of *petite* colonies using a yeast strain deficient in the Ogg1 DNA glycosylase.

## Materials and methods

### Mip1 purification and mutant construction

The open reading frame corresponding to Mip1 minus its mitochondrial targeting sequence was codon optimized for its expression in *E. coli* and purified using immobilized metal affinity chromatography (IMAC) and heparin‐affinity chromatography, as previously described [32]. Single mutants Mip1‐F763A, Mip1‐F763L, Mip1‐F763H, Mip1‐F763K, Mip1‐F763Y, and Mip1‐F763W were constructed by the Q5 mutagenesis method in an exonuclease‐deficient background that substitutes two carboxylates necessary for metal binding with alanine (D171A and E173A). This mutant is dubbed Mip1 exoˉ [59]. Mip1‐Y757C mutant was constructed in both exonuclease‐proficient and deficient backgrounds as previously described [32].

### Modeling of human DNApol γ with 8‐oxodG(*anti*):dCTP and 8‐oxodG(*syn*):dATP Mismatch

The crystal structure of human DNApol γ in complex with an incoming dNTP was superimposed with the crystal structures of T7DNAP in complex with 8‐oxodG(*anti*):dCTP and 8‐oxodG(*syn*):dATP mismatches.

### Structural modeling of Mip1 variants at the F763 position

Two wild‐type Mip1 structures (8‐oxodG(*anti*):dCTP, 8‐oxodG(*anti*):dATP) and five mutant systems for each structure were considered for the analysis. The 8‐oxodG and incoming nucleotides were modeled based on the human DNA Polymerase γ structure. Wild‐type structures were assessed for clashes and protonation using MolProbity [60], followed by the determination of protonation states from PROPKA [61]. Mutations (F763A, G763H, F763K, F763L, F763Y) were introduced into the wild‐type systems using the Dunbrack rotamer library in Chimera [62]. Ramachandran plots were used to assess potential clashes in the systems. AMBER force field parameters for 8‐oxodG were obtained from the AMBER parameter database, while parameters for dATP and dCTP were taken from a previously reported study [63, 64]. Systems were prepared with the Leap module of AMBER21 [65, 66], and all systems were minimized. The Solvent Accessible Surface Area (SASA) was calculated using visual molecular dynamics (VMD) for the residues within 5 Å of residue 763. All images were rendered with VMD [67].

### MIP1 sequence analysis

Sequence alignment including 51 amino acid sequences of mitochondrial DNA polymerases was carried out in the software geneious (version 4.8.4) [68] using the algorithm MUSCLE [69]. The sequences used in this analysis are listed in Table 4. The logo representation of the sequence alignments showcasing positions around residues 995 and 961 of human mitochondrial DNA polymerases in Fig. 1B was created with the web‐based application WebLogo [70].

**Table 4:** Amino acid sequences used in the multiple sequence alignment. The NCBI ID is shown in parentheses.

*Actinostola* sp. (KAK3722283.1)	*Equus asinus* (XP_044617408.2)
*Drosophila eugracilis* (XP_017068114.2)	*Homo sapiens* (KAI2575773.1)
*Hydra vulgaris* (XP_047140574.1)	*Aspergillus niger* (GKZ72363.1)
*Maudiozyma barnettii* (XP_041406707.1)	*Aspergillus oryzae* (OOO05376.1)
*Kluyveromyces dobzhanski*i (CDO95321.1)	*Blastomyces gilchristii* (XP_031580658.1)
*Kluyveromyces lactis* (QEU58398.1)	*Candida albicans* (XP_716738.2)
*Pichia kluyveri* (GMM46115.1)	*Coemansia* sp. (KAJ2523785.1)
*Canis lupus* (XP_038388754.1)	*Microbotryomycetes* sp. (KAK4048004.1)
*Danio rerio* (XP_001921130.3)	*Neurospora crassa* (AAD21034.1)
*Paracoccidioides brasiliensis* (XP_010760849.1)	*Penicillium antarcticum* (OQD86505.1)
*Pichia kluyveri* (GMM46115.1)	*Saccharomyces cerevisiae* (CAD6643831.1)
*Trichoderma reesei* (ETR99187.1)	*Schizosaccharomyces pombe* (KAL2313878.1)
*Trichoderma atroviride* (XP_013943976.1)	*Pocillopora meandrina* (CAH3034418.1)
*Sus scrofa* (XP_001927099.1)	*Tenebrio molitor* (XP_068908018.1)
*Acidomyces* sp. (KYG46274.1)	*Amylostereum chailletii* (KAI0319735.1)
*Castor canadensis* (XP_020021072.1)	*Coemansia* sp. (KAJ2685530.1)
*Chrysochloris asiatica* (XP_006867231.1)	*Cylindrobasidium torrendii* (KIY62530.1)
*Fusarium beomiforme* (KAF4342360.1)	*Fusarium oxysporum* (EXL48558.1)
*Lasiodiplodia mahajangana* (KAJ8131154.1)	*Lodderomyces elongisporus* (EDK46564.1)
*Marmota flaviventris* (XP_027775556.1)	*Monosporascus* sp. GIB2 (>RYP22255.1)
*Oryctolagus cuniculus* (XP_051690182.1)	*Monosporascus* sp. MC13‐8B (RYP39825.1)
*Peziza echinospora* (KAI5811077.1)	*Rhinolophus ferrumequinum* (KAF6273334.1)
*Rhizopogon vinicolor* (OAX38052.1)	*Serendipita* sp. (KAG9046149.1)
*Sinocyclocheilus grahami* (XP_016096334.1)	*Teratosphaeria destructans* (KAH9845186.1)
*Vararia minispora* (KAI0028063.1)	*Wickerhamomyces pijperi* (KAH3679690.1)
*Xylaria acuta* (KAI0455312.1)	

### Translesion bypass of 8‐oxodG assays

8‐oxodG bypassing reactions were performed in 10 mm Tris–HCl pH 8.0, 50 mm KCl, 5% glycerol, 2 mm DTT, 0.2 mg mL^−1^ bovine serum albumin (BSA), and 2 mm MgCl_2_. Reactions mixtures contained 20 nm of each of the six Mip1 mutants or wild‐type and 10 nm of ^32^P‐end‐labeled 38‐mer primer 5′‐ATT AGG AGA AAG CAC AG CGA AGT ACT AGT AAC GAC CCT‐3′ annealed with a 69‐mer template DNA 5′‐ATA TAG GGT ACC GAC TGA AGC ACT GGA CGC C**X**T AAG TCA GGG TCG TTA CTA GTA CTT CGG AGT GAC AAG‐3′ (where **X** represents G or 8‐oxodG). Reactions were preincubated for 5 min at 37 °C and initiated by adding 1 μm of dNTPs. All reactions were stopped at 3 min by the addition of an equal amount of quench buffer (95% formamide, 10 mm EDTA pH 8.0, 0.1% xylene cyanol, 0.1% bromophenol blue). The reactions were resolved on a 17% polyacrylamide‐8 m urea denaturing gels and quantified by phosphor imagery. The polymerization and full extension products were determined using the ImageJ software. In this analysis, we considered the sum of all band intensities as 100% of DNA radiolabeled to calculate the unprocessed substrate, polymerization and extension rate to describe the ability of Mip1 variants to bypass 8‐oxodG. The unprocessed substrate was determined as follows: $\text{nprocessed substrate}:\frac{I_{s}}{I_{t}}$, where *I*_s_ represents the intensity band of the DNA substrate and *I*_t_ is the sum of the intensity for each band in the lane. The polymerization rate was calculated with the following equation: $\text{olimerization rate}:\frac{I_{p}}{I_{t}}$, where *I*_p_ represents the nucleotide incorporations before 8‐oxodG or G bypass at position +8 position. The extension rate considers the nucleotide incorporation after G or 8‐oxodG bypass and is calculated as follows: $\text{xentension rate}:\frac{I_{e}}{I_{t}}$, where *I*_e_ is the sum of the intensity bands from +9 to +69 nucleotide incorporations.

### Steady‐state kinetics analysis of 8‐oxodG bypass

To obtain kinetic parameters of the different mutants of Mip1 exonuclease‐deficient, we used a dsDNA substrate containing an 8‐oxodG lesion. The substrates consisted of a 5′‐[^32^P]‐labeled 19‐mer primer 5′‐TGT TAG CAG ACA AGC CGA T‐3′ annealed to the 28‐mer template DNA 5′‐AAG AGT AC**X** ATC GGC TTG TCT GCT AAC A‐3′ where **X** is G or 8‐oxodG. Reactions were optimized so that less than 20% of the dsDNA substrate was converted into product and to assure steady‐state conditions, we varied time, enzyme, and dNTP concentrations with a fixed concentration of dsDNA substrate (10 nm). Reactions were resolved on 17% polyacrylamide–8 m urea denaturing gels and quantified by phosphor imagery. Product‐over‐substrate ratios were determined using the ImageJ software and fitted to the Michaelis–Menten equation. To obtain the kinetic parameters *K_M*, V_max, and *k_cat*, velocity and dNTP concentration were plotted using the GraphPad software as previously described [37].

### Extension of G:C, G:A, 8‐oxodG:C, and 8‐oxodG:A base pairs by Mip1 mutants

To evaluate the full extension of different Mip1 exonuclease‐deficient mutants, we used four dsDNA substrates: a 5′‐[^32^P]‐labeled 20‐mer primer 5′‐TGT TAG CAG ACA AGC CGA T**X**‐3′ annealed to a 28‐mer template 5′‐AAG AGT AC**G** ATC GGC TTG TCT GCT AAC A‐3′, where X denotes C or A to form the G:C and G:A pairs. To obtain 8‐oxodG:C and 8‐oxodG:A dsDNA substrates, we used the 28‐mer template 5′‐AAG AGT AC[**8‐oxodG**] ATC GGC TTG TCT GCT AAC A‐3′ and the radiolabeled 20‐mer primer. Reactions were prepared containing 10 nm of dsDNA substrate (G:C, G:A, 8‐oxodG:C, or 8‐oxodG:A) and each of the different Mip1 mutants. The product‐over‐substrate ratios were used to analyze the mutants' extension ability as described above.

### Kinetics analysis for dGTP incorporation opposite G:C, G:A, 8‐oxodG:C, and 8‐oxodG:A base pairs by Mip1 mutants

Reactions mixtures consisted of 10 nm of each dsDNA substrate (G:C, G:A, 8‐oxodG:C, or 8‐oxodG:A) in 10 mm Tris–HCl pH 8.0, 50 mm KCl, 5% glycerol, 2 mm DTT, 0.2 mg·mL^−1^ BSA, and 2 mm MgCl_2_. Reactions were incubated for 5 min at 37 °C before adding the catalysis buffer containing a dGTP gradient. The reactions were stopped at various times to reach less than 20% of the product by adding the quench buffer. Reactions were resolved on 17% polyacrylamide‐8 m urea denaturing gels and quantified by phosphor imaging. Kinetic constants were obtained by fitting the product: substrate ratio to the Michaelis–Menten equation as previously described above.

### Construction of the yeast mutant strains

*OGG1* and *NTG1* were disrupted in the *MIP1*‐deficient strain DWM‐5A3 (*Matα ade2‐1 leu2‐3, 112 ura3‐1 trp1‐1 his3‐11, 15 can1‐100 mip1::hphMX4*) harboring pFL38MIP1 (U*RA3* marker) through one‐step gene disruption as previously reported [39]. *ogg1::KanMX4* and *ntg1::KanMX4* cassettes were amplified from genomic DNA extracted from BY4741 *ogg1Δ* or *ntg1Δ* strains from the Euroscarf collection using oligos CTCTCCTCTTTGAATAATGTCG and CCTTGGTGACCGTTTTTTGTAG, or GGCTCGAATACGAAGTTTGG and CATATTAACAACTAGGCCTGC, respectively. Disruption was obtained by transforming the strain DWM‐5A3/pFL38MIP1 with these cassettes, selecting the deletants stain on YP (0.5% yeast extract, 1% peptone) supplemented with 2% glucose, with 200 μg·mL^−1^ geneticin and 250 μg·mL^−1^ hygromycin and checking the correctness of the disruption by PCR.

Mutations at position 763 of *MIP1* were introduced in pFL39MIP1 through the QuikChange Site‐directed mutagenesis kit (Agilent, Santa Clara, CA, USA) using specific mutagenic primers.

### Evaluation of the *petite* frequency

The effect of wild‐type and mutant Mip1 variants in the maintenance of the mtDNA in the cell population was assessed by estimating the ratio of *petite* mutant formation as previously reported [45, 46, 49]. Briefly, DWM‐5A3/pFL38MIP1 strains, with or without *OGG1* or *NTG1*, were transformed with *MIP1* gene variants cloned into pFL39 (*TRP1* marker). The plasmid shuffling was performed by 5‐fluoroorotic acid treatment, using as selection criteria the colonies that were unable to grow on plates containing synthetic complete (SC, 6.9% yeast nitrogen base without amino acids, 1 g·L^−1^ Kaiser amino acids mixture) medium without uracil supplemented with 2% glucose (indicating pFL38MIP1 loss), but able to grow on plates containing YP medium supplemented with 2% ethanol (indicating mitochondria DNA retention). Strains that accomplished the plasmid shuffling were grown on SC without tryptophan (SC‐W) plates supplemented with 2% ethanol to counter‐select the *petite* cells that could be in the population. After approximately 48 h, strains were replicated on SC‐W plates supplemented with 2% glucose and grown at 28 and 37 °C. After 24 h, a second replication was performed on DO‐W supplemented with 2% glucose and incubated again at 28 and 37 °C. After 24 h, cells were resuspended in water and approximately 200 cells were plated onto SC‐W plates supplemented with 2% ethanol and 0.3% glucose in order to distinguish *petite* and *rho* ^+^ colonies. *Petite* frequency was defined as an expression of the percentage of colonies showing the *petite* phenotype after a 5‐day incubation at 28 °C.

### qPCR on mtDNA

The effects of the Mip1 variants in maintaining the levels of mtDNA inside the cells were evaluated through qPCR. For qPCR, cells harboring *MIP1* wild‐type and mutant alleles in pFL39, with or without *OGG1*, were pregrown in YP medium supplemented with 2% ethanol at 28 °C and then inoculated and grown in YP medium supplemented with 2% glycerol and 2% ethanol at 28 or 37 °C till OD600 ≈ 1–1.5. Total DNA was extracted using the Yeast Genomic DNA Purification Kit (Avantor, Radnor, PA, USA) and quantified spectrophotometrically. A 96‐well titer plate was used to prepare the samples to be analyzed by the QuantStudio 3 Real‐Time PCR System (Thermo Fisher Scientific, Waltham, MA, USA). In each well, 15 μL of a reaction mix was loaded. The mix was made of 7.5 μL of 2X qPCRBIO SyGreen Mix Hi‐Rox (PCR Biosystems, London, England), 0.125 μm oligonucleotides for *COX1* or *ACT1* amplification, and 4 ng·μL^−1^ of total DNA. The samples were all analyzed in triplicate for each sample and target. The conditions used for the amplification were: (a) Hold phase: 2 min at 50 °C, 6 min at 95 °C; (b) Amplification phase: 40 cycles at 15 s at 95 °C, 60 s at 60 °C; (c) Melting curve analysis: 15 s at 95 °C, 60 s at a 60 °C, with an increase of 0.15 °C·s^−1^. Once the presence of only one melting curve with the same melting point for each target has been checked, the data were analyzed using the comparative ΔΔ*C*_t_ method. The Δ*C*_t_ was first calculated for each strain as *C*_t_COX1 – *C*_t_ACT1. Then the ΔΔ*C*_t_ (ΔΔ*C*_t_ = Δ*C*_t_MUT – Δ*C*_t_WT) and the 2‐ΔΔ*C*_t_ fold changes were calculated.

### Measurement of the Ery^R^ mutant frequency

The effect of the *MIP1* variants on point mutability was evaluated through measurement of the Ery^R^ mutant frequency in liquid medium through a fluctuation test by growing 10 independent colonies from two clones for each strain on YP medium supplemented with 2% ethanol and 2% glycerol for 48 h, and plating 10^8^ cells on YPAEG plates (1% yeast extract, 2% peptone, 40 μg·mL^−1^ adenine base, 3% ethanol, 3% glycerol, 25 mm H2KPO4 pH 6.5) supplemented with 3 g·L^−1^ erythromycin. Statistical analysis was performed using a Kruskal‐Wallis test followed by a *post hoc* Dunn's test.

## Conflict of interest

The authors declare no conflict of interest.

## Author contributions

NB‐T and CHT‐A wrote the initial draft of the manuscript and had seminal, experimental, and intellectual contributions. AIG and UCD executed independent experiments, data analysis, and discussion. MM‐J, PLG‐M, and CD‐Q were involved in data acquisition and analysis. GAC, TL, EB, and LGB were involved in conceptualization, funding, manuscript writing, data analysis, and discussion.

## Data Availability

The authors affirm that the data supporting the conclusions of this study can be found within the article and its supplementary materials.
